# Relationship of added sugars intakes with physiologic parameters in adults: an analysis of national health and nutrition examination survey 2001–2012

**DOI:** 10.3934/publichealth.2020037

**Published:** 2020-07-01

**Authors:** Carol E O'Neil, Theresa A Nicklas, Rabab Saab, Victor L Fulgoni

**Affiliations:** 1 LSU Agricultural Center Professor Emeritus, Baton Rouge, LA, USA; 2 USDA/ARS/CNRC, Baylor College of Medicine, Houston, TX, USA; 3 Nutrition Impact, LLC, Battle Creek, MI, USA

**Keywords:** added sugars, obesity, cardiovascular risk factors, liver enzymes, uric acid, adults

## Abstract

**Introduction:**

Consumption of added sugars (AS) has been associated with increased risk for liver disease and risk factors for cardiovascular disease. The objective of this study was to further understand the relationship of AS intake with liver enzymes and risk factors for cardiovascular disease in adults (n = 29,687) participating in the National Health and Nutrition Examination Survey (NHANES) 2001–2012.

**Methods:**

Individual usual intake (IUI) of AS was estimated using the Markov Chain Monte Carlo ratio method using two days of 24-hour dietary recalls gathered using standardized protocols. Subjects were separated into six consumption groups: 0 to <5%, 5 to <10%, 10 to <15%, 15 to <20%, 20 to <25% and ≥25% of energy as added sugars. Linear and group trends were determined using regression analyses for liver enzymes, cardiovascular risk factors, weight parameters, glucose, metabolic syndrome, and C-reactive protein. Logistic regression calculated odds ratios for these biomarkers above established risk levels (p < 0.01).

**Results:**

There was an inverse group trend association with AS IUI for lower body mass index (beta = −0.12 kg/m^2^ across AS intake groups); % overweight/obese or % obese also showed inverse group trend (−1.44, −0.77, % units across AS intake groups, respectively). Liver enzymes did not show a significant association with AS IUI. Mean plasma glucose levels (mg/dL) showed an inverse linear (beta = −0.13 mg/dL per AS intake); and group association with energy from AS IUI (beta = −0.71mg/d L across AS intake groups). There was no association of AS intake with the risk of elevated uric acid levels; however, the odds of reduced uric acid levels was 17% lower with increasing intake of energy from AS IUI in group trend analysis only (0.83; 0.72–0.95 [99^th^ CI]).

**Conclusion:**

Although it is sensible to consume AS in moderation, results suggested that higher intakes of AS were not consistently associated with physiologic parameters. Since the data were cross-sectional, they cannot be used to assess cause and effect. Thus, additional studies are warranted to confirm these findings with more rigorous study designs.

## 1. Introduction

Intake of added sugars (AS) has been associated with an increased risk of non-alcoholic fatty liver disease (NAFLD) [1]–[4]; elevated liver enzymes [5]; measures of adiposity [6]–[8]; cardiovascular disease or dyslipidemia [9]–[13], blood pressure [11]; metabolic syndrome [14],[15], diabetes [16], and hyperuricemia [17]–[19]. Other studies however have not shown health risks as a result of AS intake [20]–[26]; conflicting results from these studies, fuel the debate over AS intake on the development or exacerbation of chronic metabolic diseases. However, deriving conclusive evidence from the studies that reported positive or adverse health effects of “AS” is difficult since some studies looked only at fructose [2],[4],[8],[9],[15], or sugar sweetened beverages [1],[14],[16],[18],[26][5]–[6].

Despite the divergent results, scientists and policy makers have recently revised dietary recommendations for AS. The Institute of Medicine's (currently the National Academy of Medicine) 2002 recommendation was that no more than 25% of energy should come from added sugars. This recommendation was based primarily on nutrient dilution [27], and did not consider total energy intake or potential adverse health effects. A recent study did not show significant nutrient dilution with intakes of AS up to 25% of total energy [28]. Recently, the American Heart Association [29], World Health Organization [30], and 2015 Dietary Guidelines for Americans (DGA) [31] recommended decreasing the recommendation for added AS to <10% of energy from AS (this was based on modeling studies showing few calories remain available if a “healthy dietary pattern” is consumed). However, the 2015 DGA lists the evidence for an association between added sugars intake and cardiovascular disease as “moderate” [31] and the Evidence Analysis Library of the Academy of Nutrition and Dietetics listed the evidence for high fructose corn syrup and obesity, metabolic, and other adverse effects as Grade II (fair) [32]. The Scientific Advisory Committee on Nutrition failed to find an association between AS consumption and heart disease [33]. It is possible that a broader group to assign the responsibility of determining this recommendation needs to be established. Moreover, since the recommendation is relatively new, it's unknown if it is necessary or is unnecessarily restrictive [34]. AS intake is dropping in the U.S. [35]–[37]; while the rate of some chronic diseases or chronic disease risk factors, including metabolic syndrome in women, hypertriglyceridemia, elevated blood pressure have decreased, others including hyperglycemia and elevated waist circumference have increased [38] and obesity remains at very high levels [39],[40]. This suggests that AS are not causative for chronic disease.

As mentioned above, AS intakes have been decreasing in recent years and assessing AS relationship with physiological variables in the context of lower intakes remains relevant. The objective of this study was to further understand the relationship of increasing levels of intakes of AS as a percent of energy with physiologic parameters using a large nationally representative data set: the National Health and Nutrition Examination Survey (NHANES) 2001–2012.

## 2. Methods

### 2.1. Study overview, population, and analytic sample

An overview of the NHANES, including the purpose [41], plan and operations, sample design, weighting procedures, analytic guidelines [42],[43], and response rates and population totals [44], has been published [42]. Most NHANES data are available free of cost for scientists and the general public to download. NHANES also has available data that have limited access; these data include, but are not limited to: geographic location, the exact date of the interview, and genetic information. Data from adults 19+ years of age participating in six cycles of the NHANES (2001–2012) program of cross-sectional studies were used for these analyses (data from more recent NHANES data were not available at the time of these analyses. Those with unreliable dietary records, as judged by National Center for Health Statistics (NCHS) staff (n = 183), and pregnant or lactating females (n = 1,231) were excluded from this study; resulting in a final analytic sample of 29,687 participants. The NCHS Research Ethics Review Board approved the use of human subjects for NHANES studies [45] and additional institutional review was unnecessary [46].

### 2.2. Dietary intake assessment

Intake data were obtained from two 24-hour dietary recalls using an Automated Multiple-Pass Method (AMPM) [47] for in-person dietary interviews (Day 1) [48] and telephone interviews (Day 2) conducted 3–10 days later [49],[42]. Energy intakes were determined using the Food and Nutrient Database for Dietary Studies [50]; versions are released every two years in conjunction with the NHANES dietary data releases. AS were defined by the USDA as all sugars used as ingredients in processed and prepared foods such as breads, cakes, and soft drinks, or eaten separately or added to foods at the table [51]. AS were determined using the NHANES cycle appropriate MyPyramid Equivalents Databases for USDA Survey Food Codes from 2001–2004 [52],[53] until these were replaced by the Food Patterns Equivalents Database in 2005 for each NHANES release [54].

Individual usual intake (IUI) of AS as a percent of energy was estimated using the Markov Chain Monte Carlo (MCMC) ratio method, as implemented in v1.0 of the SAS programs published by the National Cancer Institute (NCI) [55] and is based on published work of Zhang and colleagues [56],[57]. The NCI MCMC method is used to co-model energy from sources of AS as an episodic variable and energy from sources of non-added sugars as a daily variable. The Monte Carlo dataset created contained the results of the two source variables with 500 replications per person thus providing an estimate of IUI of AS (persons with only one-day of intake were included in the analyses). Using the individual MCMC ratio AS intake, subjects were separated into six groups: 0 to <5%, 5 to <10%, 10 to <15%, 15 to <20%, 20 to <25% and ≥25% of energy intake as AS. These levels of AS intake were selected to encompass both low end dietary recommendations (i.e., <5% and <10% of calories) and reported higher end intakes influencing nutrient adequacy (i.e., >25%).

### 2.3. Demographics, anthropometric measurements, and biomarkers

Most demographic information was collected via interview using cycle appropriate questionnaires [44],[58]; the exception was alcohol intake which was obtained through the 24-hour recalls. Height, weight, and waist circumference (WC) were obtained according to NHANES protocols [59]. Body Mass Index (BMI) was calculated as body weight (kilogram) divided by height (meters) squared. Systolic and diastolic blood pressures (BP) were also determined using standard NHANES protocols [60].

The National Heart Lung and Blood Institute Guidelines [61] were used to define overweight as a BMI >25 and <29.9 and obesity as a BMI >30 kg/m^2^ [61]. Elevated WC was defined as 102 cm (males) or >88 cm (females) [61]. Laboratory methods for measuring lipids: HDL; LDL, triacylglycerols; blood glucose; C-reactive protein; liver enzymes (ALT, ALP, AST, GGT and LDH); and uric acid are available on line [62].

The National Heart Lung and Blood Institute Adult Treatment Panel III criteria was used to define metabolic syndrome as having three or more of the following risk factors: abdominal obesity, WC >102 cm (males) or >88 cm (females); hypertension, systolic blood pressure ≥130 mmHg or diastolic blood pressure ≥85 mmHg or taking anti-hypertensive medications; HDL <40 mg/dL (males) or <50 mg/dL (females); high triacylglycerols ≥150 mg/dL or taking anti-hyperlipidemic medications; high fasting glucose ≥110 mg/dL or taking insulin or other hypoglycemic agents [61].

### 2.4. Statistical analyses

Means/percentages and standard errors of demographic data by AS intake groups were determined. Regression analyses were used to assess if there was a significant difference in demographic variables across AS intake and pairwise comparisons of groups using t-tests were obtained using the SUDAAN (v 11.0). For the liver enzyme and cardiovascular risk factor levels, beta coefficients were generated from linear models regressing levels against added sugars intake as a percentage of total energy intake from AS; additionally a group trend analyses across AS intake groups was determined. Logistic regression was used to determine if the levels of added sugars intake had a lower odds ratio of having adverse physiologic outcomes. In model I, covariates used in the regression models included race/ethnicity, gender, physical activity level, alcohol intake, age, and poverty index ratio. Since there have been recent secular changes in consumption of foods and beverages with AS, a second model, included the NHANES cycle and an interaction terms of AS intake X NHANES cycle along with covariates in Model 1. (**Model II**). For Model II, there was only one variable that had a significant interaction of AS intake and NHANES cycle: HDL. Even in this case the range of difference across NHANES cycle and AS intake was quite small 52 to 53.5 mg/dL in HDL. Therefore, only results from Model I are presented. Given the differences in physical activity across added sugars intake groups, we also assessed whether there was a significant interaction of added sugars intake with physical activity. Significance was set at a p value <0.01 for all analyses and all analyses were adjusted for the complex sample design of NHANES and used appropriate subject sample weights [63].

## 3. Results

### 3.1. Demographics

When assessing group trends (Table 1), there was a significant difference in age (beta = −2.42 years; p < 0.0001), with younger individuals more likely to have a higher intake of AS than older individuals; there was also a significantly lower percentage of non-Hispanic whites consuming high levels of AS than lower levels of AS (beta = −0.82%; p = 0.0010). Individuals with higher intakes of added sugars were less likely to be sedentary (beta = −1.82%; p < 0.0001) or have moderate (beta = −1.09%; p = 0.0024) levels of physical activity; whereas, there was a higher percent of those consuming more than 25% of energy from added sugars reporting vigorous physical activity (beta = 2.56%; p < 0.0001). Individuals consuming more than 25% of energy from AS had a lower BMI (beta = −0.12 kg/m^2^; p = 0.0063) and a lower prevalence of being overweight or obese (beta = −1.44%; p < 0.0001) or being obese (beta = −0.77%; p = 0.0064) than those consuming less energy from AS. Finally, the group trend beta for energy intake was 19 kcals (p = 0.0070) with 110 kcal difference from the lowest and highest groups.

**Table 1.** Demographics of the sample of adults, 19+ years, (n = 29,687) by usual added sugars intake levels as percentage of energy.

Variable	Percent of Energy from Added Sugars Intake^1^							
	0 to <5% (n = 1,742)	5 to <10% (n = 6,897)	10 to <15% (n = 8,790)	15 to <20% (n = 6,622)	20 to <25% (n = 3,470)	≥25% (n = 2,166)	Group Trend	
	Mean ± SEM	Mean ± SEM	Mean ± SEM	Mean ± SEM	Mean ± SEM	Mean ± SEM	Beta	P*
Age	53.4 ± 0.6	49.9 ± 0.4^a^	47.1 ± 0.3^a,b^	44.7 ± 0.3^a,b,c^	42.5 ± 0.4^a,b,c,d^	41.0 ± 0.6^a,b,c,d^	−2.42	<0.0001
Gender = Male (%)	53.4 ± 1.5	50.5 ± 0.9	48.5 ± 0.7^a^	48.8 ± 0.9	46.9 ± 1.2^a^	50.0 ± 1.4	−0.74	0.0361
Ethnicity								
Non-Hispanic white (%)	74.7 ± 1.8	71.8 ± 1.5	69.7 ± 1.4^a^	70.4 ± 1.6^a^	68.8 ± 1.6^a^	69.6 ± 1.7^a^	−0.82	0.0010
Non-Hispanic black (%)	11.2 ± 1.0	11.2 ± 0.8	11.5 ± 0.8	11.1 ± 0.8	11.9 ± 0.9	11.3 ± 1.0	0.06	0.6150
Hispanic (%)	10.1 ± 1.1	11.8 ± 1.0	13.0 ± 1.0^a^	12.8 ± 1.0^a^	13.5 ± 1.2^a^	12.3 ± 1.1	0.40	0.0160
Other (%)	4.0 ± 0.6	5.2 ± 0.5	5.8 ± 0.4 ^a^	5.8 ± 0.5	5.8 ± 0.6	6.8 ± 0.8 ^a,b^	0.35	0.0235
Poverty income ratio	3.1 ± 0.1	3.0 ± 0.04	3.0 ± 0.04	2.9 ± 0.04	3.0 ± 0.1	2.9 ± 0.1	−0.03	0.0526
Physical activity								
Sedentary (%)	29.7 ± 1.2	29.9 ± 0.8	28.1 ± 0.8	26.9 ± 0.9^b^	24.5 ± 1.0^a,b,c^	23.7 ± 1.3^a,b,c^	−1.48	<0.0001
Moderate (%)	38.4 ± 1.6	36.3 ± 0.8	35.9 ± 0.8	33.0 ± 0.9^a,b^	33.8 ± 1.2	33.1 ± 1.5	−1.09	0.0024
Vigorous (%)	31.9 ± 1.6	33.8 ± 1.0	36.1 ± 0.9	40.1 ± 1.1^a,b,c^	41.7 ± 1.4^a,b,c^	43.3 ± 1.9^c^	2.56	<0.0001
Weight status								
Body mass index	28.8 ± 0.2	28.5 ± 0.1	28.2 ± 0.1	28.5 ± 0.1	28.4 ± 0.2	27.9 ± 0.2^a,b,c,d^	−0.12	0.0063
Overweight (%)	35.7 ± 1.4	34.6 ± 0.8	33.2 ± 0.8	33.2 ± 0.9	31.6 ± 1.1^a^	32.9 ± 1.5	−0.67	0.0125
Overweight or obese (%)	71.3 ± 1.6	68.6 ± 0.9	67.4 ± 0.9	66.4 ± 0.9	65.0 ± 1.2^a^	62.6 ± 1.6^a,b,c^	−1.44	<0.0001
Obese (%)	35.6 ± 1.6	34.0 ± 0.8	34.2 ± 0.9	33.2 ± 1.0	33.4 ± 1.1	29.8 ± 1.2^a,b,c^	−0.77	0.0064
Smoking current (%)	19.24 ± 1.4	22.0 ± 0.9	22.2 ± 0.8	21.6 ± 0.8	21.1 ± 1.0	21.5 ± 1.3	0.00	0.9941
Kcal mean intake	2132 ± 37	2158 ± 21	2168 ± 15	2207 ± 21	2191 ± 24	2242 ± 33	19.21	0.0070

Note: ^1^ The individual usual intake of added sugars as a percent of energy was estimated using the Markov Chain Monte Carlo ratio method as implemented in v1.0 of the SAS programs published by the NCI; Overall Group Trend Probability; letters within the table show significant differences as follows: ^a^ value is different from 0 to <5% of energy from added sugars; ^b^ value is different from 5 to <10% energy from added sugars; ^c^ value is different from 10 to <15% energy from added sugars; ^d^ value is different from 15 to <20% energy from added sugars. p < 0.01.

### 3.2. Biomarkers

There were no significant associations in either linear or group trends for liver enzyme levels or in the percentages of individuals with elevated levels of liver enzymes (Table 2) with increasing levels of energy from AS. Glucose (mg/dL) was the only cardiovascular risk factor that showed a significant linear (beta = −0.13 mg/dL; p = 0.0002) or group trend (beta = −0.71 mg/dL; p = 0.0003) (Table 3). Logistic regression showed that while there was no association of added sugars intake with the risk of elevated uric acid levels, the odds of reduced uric acid levels was 17% lower with increasing intake of energy from added sugars in group trend analysis only (0.83; 0.72–0.95 [99^th^ percentile CI]; p = 0.0083). No difference was seen for the linear trend analysis for reduced uric acid levels; no other differences were found (Table 4). Only four variables (% elevated diastolic BP, LDL, alkaline phosphatase, and % elevated LDL) had an indication (p < 0.10) of the existence of an interaction of added sugars intake and physical activity (Supplemental Table 1). The % elevated diastolic BP and % elevated LDL increased at a faster rate across added sugars intakes for those sedentary as compared to those with moderate activity while LDL increased at a faster rate across added sugars intakes for those sedentary as compared to those with vigorous activity and alkaline phosphatase decreased across added sugars intakes for those sedentary but increased slightly for those with vigorous activity.

**Table 2.** Liver enzymes of adults (19+ years of age) grouped by percent intake of added sugars: NHANES 2001–2012.

Variable	Percent Energy from Added Sugars^1^									
	0 to <5%	5 to <10%	10 to <15%	15 to <20%	20 to <25%	≥25%	Linear Trend		Group Trend	
	LSM ± SEM	LSM ± SEM	LSM ± SEM	LSM ± SEM	LSM ± SEM	LSM ± SEM	Beta	P	Beta	P
ALP (U/L)	68.0 ± 0.7	67.4 ± 0.4	67.5 ± 0.4	67.5 ± 0.5	67.4 ± 0.6	67.0 ± 0.6	−0.03	0.2587	−0.10	0.4753
ALP elevated^2^	0.7 ± 0.3	0.9 ± 0.2	0.8 ± 0.1	0.9 ± 0.2	0.7 ± 0.2	0.7 ± 0.2	0.00	0.8519	−0.01	0.7648
ALT (U/L)	27.60 ± 1.51	25.4 ± 0.3	25.9 ± 0.4	25.8 ± 0.3	26.4 ± 0.5	24.8 ± 0.5	−0.03	0.2687	−0.12	0.4113
ALT elevated^3^	7.9 ± 1.0	6.5 ± 0.4	7.2 ± 0.4	7.1 ± 0.4	7.8 ± 0.7	5.6 ± 0.8	−0.02	0.4483	−0.05	0.7582
AST (U/L)	26.8 ± 1.1	25.7 ± 0.3	26.1 ± 0.3	26.3 ± 0.4	27.2 ± 0.6	26.5 ± 1.0	0.04	0.2512	0.16	0.3128
AST elevated^4^	6.9 ± 1.0	6.6 ± 0.5	6.9 ± 0.4	7.1 ± 0.4	6.8 ± 0.6	5.6 ± 0.6	−0.03	0.2824	−0.10	0.5337
GGT (U/L)	28.7±1.0	27.7 ± 0.6	29.3 ± 0.6	28.2 ± 0.6	29.9 ± 1.1	27.3 ± 1.1	0.00	0.9512	0.10	0.6724
GGT elevated^5^	5.8 ± 0.9	4.5 ± 0.4	5.0 ± 0.3	4.9 ± 0.4	6.5 ± 0.7	4.5 ± 0.6	0.01	0.6578	0.14	0.2607
LDH (U/L)	128.3 ± 1.0	128.2 ± 0.6	128.3 ± 0.5	128.4 ± 0.5	129.3 ± 0.6	127.9 ± 0.8	0.02	0.5158	0.10	0.5205
LDH elevated^6^	0.1 ± 0.1	0.2 ± 0.1	0.2 ± 0.1	0.1 ± 0.02	0.3 ± 0.1	0.1 ± 0.1	0.00	0.8490	0.00	0.9736

Note: Covariates: age, gender, ethnicity, poverty index ratio, physical activity level, and alcohol consumption. Abbreviations: ALP = alkaline phosphatase; ALT = alanine aminotransferase; AST = aspartate aminotransferase; GGT = gamma-glutamyl transferase; LDH = Lactate dehydrogenase. ^1^ Markov chain Monte Carlo (MCMC) ratios as implemented in v1.0 of the SAS programs published by the National Cancer Institute; p < 0.01. ^2^ ALP High: alkaline phosphatase (U/L) >140. ^3^ ALT High: alanine aminotransferase (U/L) >38 (female) or >50 (male). ^4^ AST High: aspartate aminotransferase (U/L) >34 (female) or >40 (male). ^5^ GGT High: gamma glutamyl transferase (U/L) >85 (male) >55 (female). ^6^ LDH High: lactate dehydrogenase (U/L) >280.

**Table 3.** Cardiovascular risk factors and Metabolic Syndrome (LSM ± SE) and % with elevated levels of cardiovascular risk factors categorized by % of energy of added sugars intake in adults: NHANES 2001–2012.

Variable	Percent Energy of Added Sugars Intake^1^										
	n	0 to <5%	5 to <10%	10 to <15%	15 to <20%	20 to <25%	≥25%	Linear trend		Group trend	
		LSM ± SE	LSM ± SE	LSM ± SE	LSM ± SE	LSM ± SE	LSM ± SE	Beta	P	Beta	P
BP (%) elevated^2^	26,780	40.1 ± 1.6	44.0 ± 0.8	42.6 ± 0.7	43.4 ± 0.9	43.0 ± 1.0	43.4 ± 1.5	0.03	0.6562	0.15	0.6267
BP systolic^3^	26,554	122.0 ± 0.5	122.6 ± 0.4	122.5 ± 0.3	122.6 ± 0.3	122.2 ± 0.3	122.3 ± 0.5	0.00	0.9313	−0.03	0.7609
BP systolic (%) elevated^4^	26,780	38.7 ± 1.7	41.0 ± 0.8	40.3 ± 0.7	40.7 ± 0.8	40.6 ± 1.0	41.8 ± 1.4	0.05	0.4078	0.24	0.4114
BP diastolic^3^	26,418	71.6 ± 0.4	71.5 ± 0.3	71.2 ± 0.2	71.5 ± 0.3	71.0 ± 0.4	71.0 ± 0.5	−0.03	0.0922	−0.12	0.1289
BP diastolic (%) elevated^5^	26,780	29.5 ± 1.5	32.4 ± 0.9	32.0 ± 0.7	33.6 ± 0.9	32.9 ± 0.8	33.5 ± 1.5	0.11	0.0851	0.53	0.0856
LDL-cholesterol (mg/dL)	12,324	115.0 ± 1.6	116.6 ± 1.0	116.2 ± 0.8	117.1 ± 0.8	117.2 ± 1.1	115.2 ± 1.5	0.01	0.8802	0.09	0.7416
LDL (%) elevated^6^	12,525	74.2 ± 1.7	75.2 ± 1.0	73.6 ± 1.1	75.3 ± 0.9	75.3 ± 1.4	72.42 ± 2.1	−0.03	0.6319	−0.12	0.7279
HDL-cholesterol (mg/dL)	26,081	51.9 ± 0.4	52.8 ± 0.3	52.4 ± 0.3	53.3 ± 0.3	52.8 ± 0.4	53.3 ± 0.4	0.03	0.0568	0.21	0.0218
HDL-cholesterol reduced (%)^7^	26,314	45.3 ± 1.6	41.1 ± 0.8	41.6 ± 0.8	39.7 ± 0.8	40.8 ± 1.1	40.1 ± 1.5	−0.09	0.1017	−0.63	0.0288
Triacylglycerides (mg/dL)	12,753	132.7 ± 4.9	141.3 ± 3.6	140.0 ± 3.0	133.3 ± 2.2	137.5 ± 4.4	137.7 ± 4.2	−0.10	0.6144	−0.78	0.4726
Triacylglycerides elevated^8^ (%)	12,870	40.0 ± 2.4	38.3 ± 1.3	39.2 ± 0.9	36.4 ± 1.0	39.4 ± 1.6	38.4 ± 2.1	−0.05	0.5472	−0.22	0.6398
WC (cm)	26,471	98.0 ± 0.6	97.5 ± 0.3	97.6 ± 0.3	97.6 ± 0.3	97.5 ± 0.4	96.7 ± 0.4	−0.03	0.1121	−0.13	0.1551
WC (%) elevated^9^	26,471	54.0 ± 1.7	53.4 ± 1.0	52.06 ± 0.9	53.3 ± 1.0	51.8 ± 1.1	49.6 ± 1.4	−0.13	0.0380	−0.60	0.0513
Glucose, plasma (mg/dL)	12,870	105.7 ± 1.8	104.3 ± 0.7	103.5 ± 0.5	102.46 ± 0.6	102.1 ± 0.6	102.3 ± 0.9	−0.13	0.0002	−0.71	0.0003
Glucose (%) elevated^10^	14,269	49.9 ± 2.0	50.0 ± 1.2	50.1 ± 1.2	48.0 ± 1.3	46.5 ± 1.7	46.5 ± 2.4	−0.20	0.0184	−0.98	0.0218
Metabolic syndrome^11^	19,245	46.0 ± 1.8	44.6 ± 1.0	43.5 ± 0.8	43.6 ± 1.0	44.5 ± 1.2	43.1 ± 1.5	−0.07	0.3132	−0.30	0.3786
CRP (mg/dL)	21,947	0.4 ± 0.04	0.4 ± 0.01	0.4 ± 0.01	0.4 ± 0.01	0.4 ± 0.01	0.4 ± 0.02	0.00	0.4843	−0.01	0.2351
CRP (%) elevated^12^	21,947	0.8 ± 0.4	1.2 ± 0.2	1.5 ± 0.2	1.0 ± 0.1	1.0 ± 0.2	1.2 ± 0.3	0.00	0.8391	−0.04	0.6119

Note: Covariates: age, gender, ethnicity, poverty index ratio, poverty index ratio, physical activity level, and alcohol consumption. Abbreviations: BP = blood pressure; WC = Waist Circumference; CRP = C-reactive protein. ^1^ The usual intake of added sugars as a percent of energy was estimated using the Markov Chain Monte Carlo ratio method as implemented in v1.0 of the SAS programs published by the National Cancer Institute. ^2^ elevated blood pressure: ≥130 mmHg systolic blood pressure or diastolic blood pressure ≥85 mmHg; ^3^ mean mm Hg; ^4^ elevated systolic blood pressure: ≥130 mmHg; ^5^ elevated diastolic blood pressure: ≥85 mmHg; ^6^ elevated LDL: >100 mg/dL; ^7^ reduced serum HDL: <40 mg/dL (males), <50 mg/dL (females); ^8^ elevated serum triglycerides: ≥150 mg/dL; ^9^ elevated waist circumference: ≥102 cm (males), ≥88 cm (females); ^10^ elevated fasting plasma or serum glucose: ≥100mg/dL; ^11^ metabolic syndrome was defined using the NHLBI Adult Treatment Panel III criteria; ^12^ elevated C-reactive protein = 3mg/dL.

**Table 4.** Likelihood^1^ of having elevated liver enzymes, cardiovascular risk factors, and metabolic syndrome, and altered uric acid levels categorized by % of energy of added sugars intake^2^ in adults: NHANES 2001–2012.

Variable	Percent Energy of Added Sugars Intake								
	5 to <10%	10 to <15%	15 to <20%	20 to <25%	≥25%	Linear trend		Group trend	
	OR (LCL,UCL)	OR (LCL,UCL)	OR (LCL,UCL)	OR (LCL,UCL)	OR (LCL,UCL)	OR (LCL,UCL)	P	OR (LCL,UCL)	P
ALT elevated^3^	0.80 (0.58,1.10)	0.89 (0.66,1.21)	0.88 (0.65,1.21)	0.97 (0.70,1.34)	0.69 (0.46,1.03)	1.00 (0.99,1.01)	0.4374	0.99 (0.94,1.04)	0.7442
AST elevated^4^	0.95 (0.67,1.34)	0.99 (0.72,1.36)	1.03 (0.75,1.42)	0.97 (0.68,1.39)	0.79 (0.55,1.14)	0.99 (0.99,1.00)	0.2898	0.98 (0.94,1.04)	0.5401
ALP elevated^5^	1.18 (0.64,2.21)	1.11 (0.57,2.15)	1.17 (0.58,2.35)	0.99 (0.43,2.23)	0.91 (0.40,2.07)	1.00 (0.97,1.02)	0.7099	0.97 (0.86,1.10)	0.6392
GGT elevated^6^	0.77 (0.52,1.12)	0.85 (0.62,1.17)	0.84 (0.60,1.19)	1.15 (0.81,1.64)	0.75 (0.48,1.17)	1.00 (0.99,1.01)	0.6565	1.03 (0.98,1.09)	0.2554
LDH elevated^7^	2.03 (0.27,15.12)	1.96 (0.28,13.97)	1.03 (0.15,7.09)	3.06 (0.39,23.86)	0.88 (0.08,9.51)	1.00 (0.96,1.05)	0.8964	1.00 (0.77,1.30)	0.9835
BP elevated^8^	1.25 (1.03,1.53)	1.16 (0.95,1.42)	1.22 (0.99,1.50)	1.19 (0.97,1.47)	1.21 (0.95,1.55)	1.00 (0.99,1.01)	0.5744	1.01 (0.97,1.05)	0.5436
BP systolic elevated^9^	1.15 (0.94,1.41)	1.10 (0.89,1.36)	1.13 (0.92,1.39)	1.13 (0.90,1.41)	1.21 (0.94,1.54)	1.00 (1.00,1.01)	0.3969	1.02 (0.98,1.05)	0.3948
BP diastolic elevated^10^	1.16 (0.98,1.39)	1.13 (0.94,1.37)	1.24 (1.03,1.49)	1.18 (0.98,1.42)	1.21 (0.94,1.55)	1.01 (1.00,1.01)	0.1633	1.03 (0.99,1.07)	0.1592
LDL elevated^11^	1.06 (0.81,1.39)	0.97 (0.75,1.26)	1.08 (0.84,1.39)	1.08 (0.83,1.41)	0.95 (0.68,1.32)	1.00 (0.99,1.01)	0.9185	1.00 (0.96,1.04)	0.9915
HDL reduced^12^	0.84 (0.72,0.97)	0.85 (0.74,0.98)	0.79 (0.68,0.91)	0.82 (0.69,0.98)	0.80 (0.66,0.97)	1.00 (0.99,1.00)	0.0896	0.97 (0.95,1.00)	0.0249
Triacylglycerides elevated^13^	0.93 (0.73,1.17)	0.97 (0.78,1.19)	0.84 (0.66,1.07)	0.97 (0.73,1.29)	0.92 (0.68,1.26)	1.00 (0.99,1.01)	0.5144	0.99 (0.95,1.03)	0.6011
Glucose elevated^14^	1.01 (0.81,1.25)	1.01 (0.81,1.26)	0.92 (0.72,1.16)	0.86 (0.68,1.08)	0.85 (0.63,1.16)	0.99 (0.98,1.00)	0.0216	0.96 (0.92,0.99)	0.0246
Metabolic Syndrome^15^	0.94 (0.76,1.16)	0.89 (0.72,1.11)	0.90 (0.72,1.12)	0.94 (0.73,1.21)	0.86 (0.66,1.12)	1.00 (0.99,1.00)	0.3429	0.98 (0.95,1.02)	0.4035
C-reactive protein elevated^16^	1.41 (0.59,3.39)	1.78 (0.73,4.35)	1.16 (0.49,2.74)	1.20 (0.51,2.80)	1.34 (0.46,3.87)	1.00 (0.97,1.02)	0.8331	0.97 (0.86,1.09)	0.6032
Waist circumference elevated^17^	0.98 (0.83,1.15)	0.92 (0.77,1.09)	0.97 (0.82,1.15)	0.91 (0.76,1.09)	0.83 (0.69,0.99)	0.99 (0.99,1.00)	0.0437	0.97 (0.95,1.00)	0.0580
Uric acid elevated^18^	0.87 (0.70,1.08)	0.90 (0.72,1.12)	0.89 (0.70,1.12)	1.12 (0.89,1.42)	0.95 (0.73,1.24)	1.00 (1.00,1.01)	0.1347	1.03 (0.99,1.07)	0.1186
Uric acid reduced^19^	1.07 (0.38,3.00)	0.65 (0.25,1.72)	0.97 (0.38,2.48)	0.43 (0.15,1.20)	0.30 (0.09,0.96)	0.97 (0.94,1.00)	0.0223	0.83 (0.72,0.95)	0.0083

Note: Covariates: age, gender, ethnicity, poverty index ratio, physical activity level, and alcohol consumption. ^1^ Logistic regression with 99% confidence intervals with the <5% added sugars intake group as the referent group; ^2^ the usual intake of added sugars as a percent of energy was estimated using the Markov Chain Monte Carlo ratio method as implemented in v1.0 of the SAS programs published by the National Cancer Institute; ^3^ elevated ALT ≥38 U/L (females); >50 U/L (males); ^4^ elevated AST >34 U/L (females); >40 (males); ^5^ ALP >140 U/L; ^6^ GGT >55 U/L (females); >85 U/L (males); ^7^ LDH >280 U/L; ^8^ elevated blood pressure: ≥130 mmHg systolic blood pressure or diastolic blood pressure ≥85 mmHg; ^9^ elevated systolic blood pressure : ≥130 mmHg; ^10^ elevated diastolic blood pressure: ≥85 mmHg; ^11^ elevated LDL: >100 mg/dL; ^12^ reduced serum HDL: <40 mg/dL (males), <50 mg/dL (females); ^13^ elevated triglycerides: ≥150 mg/dLL; ^14^ elevated serum glucose: ≥100 mg/dL; ^15^ Metabolic Syndrome was defined using the NHLBI Adult Treatment Panel III criteria; ^16^ elevated C-reactive protein: >3 mg/dL; ^17^ elevated waist circumference: ≥102 cm (males), ≥88 cm (females); ^18^ elevated uric acid levels (mg/dL): >7.2 (male) >6.1 (female); ^19^ reduced uric acid levels (mg/DL): <3.4 (male), <2.4 (female). Abbreviations: LCL = lower confidence level; UCL = upper confidence level; ALT = alanine aminotransferase; AST = aspartate aminotransferase; ALP = alkaline phosphatase; GGT = gamma-glutamyl transferase; LDH = Lactate dehydrogenase; BP = blood pressure.

## 4. Discussion

This study showed that there were a number of demographic differences in the population across increasing levels of AS intake, notably in age with younger individuals consuming more added sugars than older individuals and in physical activity categories. Despite a slight increase in energy intake across percent energy from AS, BMI, and the percent overweight and/or obese and percent obese were lower at the highest categories of AS intake, though these results were unadjusted for other covariates. This paradoxical association of AS intake with a lower BMI and lower percent overweight and/or obese could be explained by the more vigorous physical activity reported in those with high intakes of AS. The clinical significance of the differences in BMI are unclear and warrant further study.

There were few differences in physiologic parameters across increasing levels of AS intake, including none for liver enzymes; among the cardiovascular risk factors, only an inverse linear and group trend was found for glucose levels (mg/dL). Logistic regression showed that the odds of reduced uric acid levels were 17% lower with increasing intake of AS for group trend analysis only. All other results suggested no association of usual AS intake with risk factors.

There was a small, but significant difference, in mean BMI in the group consuming more than 25% of energy from AS when compared with all groups consuming less than 25% of energy from AS. There were also small, but significant differences in the percentage of the population of overweight or obese or obese individuals at the higher levels of intake (≥25% of energy) from AS. This is counterintuitive to what one might expect, based on the literature [6]–[8]; However, it could be the result of the higher percentage of those reporting vigorous levels of physical activity or underreporting in individuals consuming higher levels of AS. The total energy intake of all six groups of AS intake was consistent with what is reported in What We Eat in America [64] and is consistent with recommended energy requirements for most adult age groups [51]. Additional studies are needed to determine the reasons for these results.

AS consumption, particularly in the form of fructose alone or as a component of sugar sweetened beverages, has been associated with a number of metabolic derangements, including inflammation of the liver and the development or exacerbation of NAFLD [65],[66]. Standard hepatic function tests include, but are not limited to, the enzymes assessed in this study: ALT, ALP, AST, GGT, and LDH. Although all are measured when liver disease is suspected, elevated levels can also indicate other health issues, including cardiovascular disease and other inflammatory diseases [67]. Significant liver disease may not be associated with increased liver enzymes, but, it should be noted that increased levels of ALT and AST are good predictors of NAFLD [68]. Some liver enzymes, notably GGT [69], are also usually elevated in NAFLD. Our results support those of a systematic review and meta-analysis which suggested that AS intake was not associated with derangements of liver enzymes [65]. That none of the liver enzymes (absolute values or percent elevated values) assessed in this study showed significant linear or group trends and that the odds ratios did not show a significant likelihood of the liver enzymes increasing with increasing intake of AS intake suggests, but does not confirm, there may be no effect on liver injury. The lack of any associations was also shown for the age groups 19–50 years and 50+ years and for both genders when analyzed separately (data not shown).

There has been a paucity of studies that have looked at liver enzymes and AS intake; Shimomy et al. [5] showed that serum ALT and AST levels were seen in healthy pre-menopausal women with habitual or moderate intake of sugar sweetened beverages. In that study, “total fructose was calculated as the sum of free fructose and half the intake of added sugar” which may have over-estimated the fructose intake. In their meta-analysis, Chiu et al. [65], found that ALT was elevated with high intakes of fructose, but the authors concluded that the effect may have been due to excess energy intake. Failure to control for total energy intake is a limitation of some of these studies [70].

That none of the cardiovascular risk factors, with the exception of the inverse association of AS intake with fasting plasma glucose levels, were significantly different across the groups of increasing percent energy of AS intake was surprising. Welch et al. [12], using NHANES data (1999–2006) showed that adjusted mean HDL levels significantly decreased with increasing intake of AS and the geometric mean triacylglyceride levels and, in women only, LDL levels increased. The covariates used in that study and ours were similar; However, Welch et al. [12], also used history of attempted weight loss in the previous year, and weight change. The latter two covariates have been shown to effect HDL levels [71]; However, were not used in this study due to the wider array of variables examined herein. Two other potential reasons for the differences seen in those data compared with the data in this study is that they used one day intake only, as opposed to the IUI used in this study, and they used a less conservative level of significance (p < 0.05).

In some [72], but not all studies [20], high uric acid levels have been associated with AS intake, especially fructose and high fructose corn syrup, high levels have, in turn, been associated with elevated levels of ALT and GGT [73]. None of the groups of AS intake by percent energy in this study had elevated uric acid levels; as with the liver enzymes, none of the age groups and neither gender, when analyzed separately, showed any differences. However, a lower odds ratio of having reduced uric acid levels in the highest added sugars intake was found. The reasons are not clear. In one study [72] where hyperuricaemia was associated with consumption of fructose, it appears that the effect is transient, since most of the participants in this study were fasting it may be that the time lapse between consumption of AS and collection of serum had mitigated the effects. Further studies are needed to determine why the group trend for reduced uric acid was associated with AS intake.

This study had a number of strengths, notably the large sample size of a nationally representative dataset. The NHANES has a large representative sample and uses validated methods to collect two 24-hour dietary recalls, which can then be used to determine IUI [55],which in turn reduces measurement error. The NHANES also uses measured anthropometrics and laboratory values.

This study also had a number of limitations. Data used in this study did not include the more recent NHANES 2013–2016 cycles because they were not available at the time the study was conducted. However, given the large sample size (over 29,000 subjects) from the 2001–2012 cycles was clearly large enough to complete the study. As with all cross-sectional studies cause and effect relationships could not be determined. Twenty-four hour dietary recalls are memory-dependent and there is an underreporting bias in energy intake that is higher in overweight and obese individuals, and women [74]. However, the degree of underreporting with the AMPM of taking 24-hour dietary recalls is lower than seen with other intake instruments [75]. Individuals may unintentionally misreport dietary intake; For example, they may state that they consumed 100% fruit juice, which has no AS, but actually consumed a fruit drink, which does. Variation in AS content of foods and beverages across nutrient databases is also possible and may lead to classification error [76],[77]. Errors like these could lead to misclassification of individuals into groups. Linear regression analyses were done to limit any potential effect of misclassification. As with any study involving analysis of large epidemiologic study data, residual confounding may occur. Finally, the results could reflect the influence of other foods consumed/not consumed throughout the day among the AS intake groups.

## 5. Conclusion

Higher intakes of AS were not consistently associated with liver enzymes, cardiovascular disease biomarkers, including blood pressure, lipid levels, and glucose levels; or elevated uric acid levels. Data suggest that current recommendations for AS intake may be overly restrictive, at least for some people, and restrictive AS intake may lead to some Americans not meeting recommended micronutrient intakes [78]. Since the data were cross-sectional, they cannot be used to determine cause and effect. Thus, additional studies are warranted to confirm these findings with more rigorous study designs, especially if AS intakes continue to change over time.
