# Supplementary material for: Relationship of added sugars intakes with physiologic parameters in adults: an analysis of national health and nutrition examination survey 2001–2012
Source: AIMS Public Health. 2020 Jul 1;7(3):450–68. doi: 10.3934/publichealth.2020037 (PMC7505778; doi:10.3934/publichealth.2020037)
Supplement: Supplementary file 1 [file publichealth-07-03-037-s001.pdf]

Public Health

AIMS Public Health, 7(3): 450–468.
DOI: 10.3934/publichealth.2020037
Received: 02 June 2020
Accepted: 22 June 2020
Published: 01 July 2020

http://www.aimspress.com/journal/aimsph

Research article

Relationship of added sugars intakes with physiologic parameters in adults: an analysis of national health and nutrition examination survey 2001–2012

Carol E O'Neil[1], Theresa A Nicklas[2,*], Rabab Saab[2] and Victor L Fulgoni[3]

[1] LSU Agricultural Center Professor Emeritus, Baton Rouge, LA, USA
[2] USDA/ARS/CNRC, Baylor College of Medicine, Houston, TX, USA
[3] Nutrition Impact, LLC, Battle Creek, MI, USA

* **Correspondence:** Email: tnicklas@bcm.edu; Tel: 713-798-7087.

Supplementary

Supplemental Table 1. Assessment of interaction of added sugars intake and physical activity[1].

Variable	β1[2]	SE	P[3] β1 = 0	β2	SE	P β2 = 0	β3	SE	P β3 = 0	P[4] β1 = β2 = β3	P[5] β1 = β2	P β1 = β3	P β2 = β3
BP diastolic elevated (%)	0.299	0.103	0.0047	−0.056	0.102	0.5845	0.164	0.084	0.0547	0.0201	0.0054	0.2791	0.1028
LDL-cholesterol (mg/dL)	0.268	0.101	0.0095	0.033	0.116	0.7790	−0.150	0.113	0.1871	0.0362	0.1422	0.0106	0.2648
Alkaline phosphatase (U/L)	−0.111	0.044	0.0143	−0.015	0.038	0.6876	0.031	0.042	0.4687	0.0404	0.1010	0.0123	0.4053
LDL elevated (%)	0.197	0.122	0.1096	−0.173	0.127	0.1769	−0.075	0.129	0.5610	0.0730	0.0276	0.1316	0.5960
Alkaline phosphatase elevated (%)	−0.005	0.020	0.8030	−0.015	0.013	0.2663	0.014	0.011	0.1951	0.2184	0.6726	0.3746	0.0881
ALT elevated (%)	0.005	0.072	0.9411	−0.064	0.058	0.2744	0.000	0.042	0.9960	0.6412	0.4591	0.9494	0.3745
ALT: SI (U/L)	−0.026	0.084	0.7592	−0.079	0.048	0.1017	0.018	0.033	0.5869	0.2786	0.5896	0.6202	0.1157
AST elevated (%)	−0.043	0.059	0.4674	−0.098	0.051	0.0602	0.037	0.045	0.4145	0.1312	0.5256	0.2239	0.0644
AST: SI (U/L)	−0.006	0.046	0.9003	−0.023	0.029	0.4213	0.031	0.019	0.1031	0.2507	0.7669	0.4310	0.1416
BP diastolic (mean rdg mm hg)	0.022	0.027	0.4073	−0.029	0.025	0.2542	−0.046	0.025	0.0626	0.1571	0.1308	0.0704	0.6104
BP elevated (%)	0.120	0.105	0.2580	−0.087	0.094	0.3605	0.113	0.085	0.1893	0.1508	0.1237	0.9576	0.0891
BP systolic (mean rdg mm hg)	−0.017	0.050	0.7258	0.000	0.033	0.9886	0.020	0.029	0.4901	0.8025	0.7586	0.5330	0.6465
BP systolic elevated (%)	0.128	0.105	0.2248	−0.005	0.094	0.9567	0.092	0.079	0.2430	0.5440	0.3283	0.7965	0.3903
C-reactive protein (mg/dL)	0.000	0.002	0.8291	0.001	0.002	0.5820	−0.001	0.002	0.6574	0.6904	0.6210	0.8706	0.3967
C-reactive protein elevated (%)	0.009	0.022	0.6816	0.005	0.028	0.8563	−0.008	0.014	0.5657	0.7856	0.8911	0.5133	0.6436
GGT elevated (%)	0.009	0.045	0.8482	0.014	0.056	0.8028	0.012	0.037	0.7445	0.9968	0.9438	0.9499	0.9800
GGT: SI (U/L)	0.017	0.067	0.7971	0.059	0.085	0.4877	−0.051	0.064	0.4285	0.5390	0.6991	0.4425	0.2992
Glucose elevated (%)	−0.112	0.151	0.4600	−0.270	0.134	0.0461	−0.167	0.122	0.1717	0.7262	0.4356	0.7658	0.5747
Glucose, plasma (mg/dL)	−0.193	0.082	0.0210	−0.239	0.081	0.0039	−0.064	0.051	0.2116	0.1432	0.7028	0.1789	0.0828
HDL reduced (%)	−0.151	0.094	0.1125	−0.145	0.098	0.1421	0.064	0.089	0.4740	0.1680	0.9655	0.0818	0.1451
HDL-cholesterol (mg/dL)	0.031	0.033	0.3592	0.027	0.030	0.3626	0.027	0.030	0.3662	0.9952	0.9357	0.9311	0.9943
LDH (U/L)	−0.001	0.059	0.9823	0.004	0.047	0.9348	0.048	0.040	0.2311	0.6624	0.9439	0.4949	0.4241

Continued on next page

Variable	β1[2]	SE	P[3] β1 = 0	β2	SE	P β2 = 0	β3	SE	P β3 = 0	P[4] β1 = β2 = β3	P[5] β1 = β2	P β1 = β3	P β2 = β3
LDH elevated (%)	−0.003	0.009	0.7739	−0.004	0.003	0.2019	0.002	0.003	0.4160	0.1996	0.8523	0.6019	0.0754
Metabolic syndrome (%)	−0.084	0.102	0.4105	−0.143	0.096	0.1394	0.061	0.095	0.5194	0.3276	0.6554	0.3188	0.1393
Triglycerides (mg/dL)	−0.461	0.372	0.2190	−0.359	0.307	0.2447	0.553	0.415	0.1861	0.1266	0.8400	0.0655	0.0822
Triglycerides elevated (%)	−0.160	0.135	0.2394	−0.090	0.153	0.5553	0.044	0.140	0.7515	0.5688	0.7123	0.2895	0.5360
Uric acid elevated (%)	−0.009	0.084	0.9110	0.094	0.066	0.1601	0.129	0.082	0.1172	0.5082	0.3708	0.2631	0.7489
Uric acid reduced (%)	−0.023	0.018	0.2040	−0.023	0.019	0.2295	−0.024	0.015	0.1298	0.9999	0.9938	0.9937	0.9881
Waist circum elevated (%)	0.017	0.086	0.8434	−0.121	0.087	0.1678	−0.111	0.080	0.1681	0.4769	0.2689	0.3144	0.9370
Waist Circumference (cm)	−0.007	0.012	0.5718	−0.007	0.014	0.6299	−0.014	0.011	0.2258	0.8971	0.9901	0.6796	0.7089

Note: [1] SAS PROC SURVEYREG was used with strata and primary sampling units to assess interaction of added sugars intake with physical activity level (1 = sedentary, 2 = moderate, 3 = vigorous). β1, β2, β3 represent the beta coefficient for added sugars intake for each respective level of physical activity. Contrast statements are used to test hypotheses involving linear combinations of β1, β2, β3; [2] Beta coefficient for added sugars intake in those sedentary (physical activity level = 1); β2 and β3 are beta coefficient for added sugars intake in those with moderate (physical activity level = 2) and vigorous physical activity (physical activity level =3), respectively; [3] P-value for testing hypothesis β1 = 0; other p-values provided for testing whether β2 = 0 and whether β3 = 0; [4] β1 = β2 = β3 = 0 tests the hypothesis that there is no linear trend across added sugars intake for any of the physical activity levels; $p < 0.10$ was used as an indication of a possible interaction of added sugars intake and physical activity; [5] P-value for testing hypothesis that beta coefficients are equal.

AIMS Press

© 2020 the Author(s), licensee AIMS Press. This is an open access article distributed under the terms of the Creative Commons Attribution License (http://creativecommons.org/licenses/by/4.0)
